# Modulation of mRNA 3′-End Processing and Transcription Termination in Virus-Infected Cells

**DOI:** 10.3389/fimmu.2022.828665

**Published:** 2022-02-10

**Authors:** Aarthi Vijayakumar, Annsea Park, Joan A. Steitz

**Affiliations:** ^1^Department of Molecular Biophysics and Biochemistry, Yale University, New Haven, CT, United States; ^2^Department of Immunobiology, Yale University, New Haven, CT, United States

**Keywords:** mRNA 3'-end processing, transcription termination, virus-infected cells, read-through transcription, downstream-of-gene transcripts

## Abstract

Eukaryotic mRNA 3´-end processing is a multi-step process beginning with pre-mRNA transcript cleavage followed by poly(A) tail addition. Closely coupled to transcription termination, 3´-end processing is a critical step in the regulation of gene expression, and disruption of 3´-end processing is known to affect mature mRNA levels. Various viral proteins interfere with the 3´-end processing machinery, causing read-through transcription and altered levels of mature transcripts through inhibition of cleavage and polyadenylation. Thus, disruption of 3´-end processing contributes to widespread host shutoff, including suppression of the antiviral response. Additionally, observed features of read-through transcripts such as decreased polyadenylation, nuclear retention, and decreased translation suggest that viruses may utilize these mechanisms to modulate host protein production and dominate cellular machinery. The degree to which the effects of read-through transcript production are harnessed by viruses and host cells remains unclear, but existing research highlights the importance of host 3´-end processing modulation during viral infection.

## Highlights

- Viruses disrupt host transcription termination by interfering with the mRNA 3´-end processing machinery.
- This disruption leads to the production of read-through transcripts called Downstream-of-Gene (DoG) transcripts that include the upstream mRNA sequence but are retained in the nucleus and are therefore non-coding.
- Influenza A virus protein NS1 and herpes simplex virus-1 protein ICP27 inhibit canonical cellular mRNA cleavage, polyadenylation, or both through interference with CPSF subunits.
- Disruption of transcription termination in virus-infected cells occurs on a genome-wide scale and affects the antiviral response.
- Viruses selectively disrupt host 3´-end processing while viral transcription and 3´-end processing remain intact.
- Read-through transcription has a multitude of effects, including downregulation of genes that experience readthrough and inhibition of downstream genes *via* read-in transcription.

## Canonical Eukaryotic 3´-end Processing Consists of Cleavage and Polyadenylation

**Figure 1:**
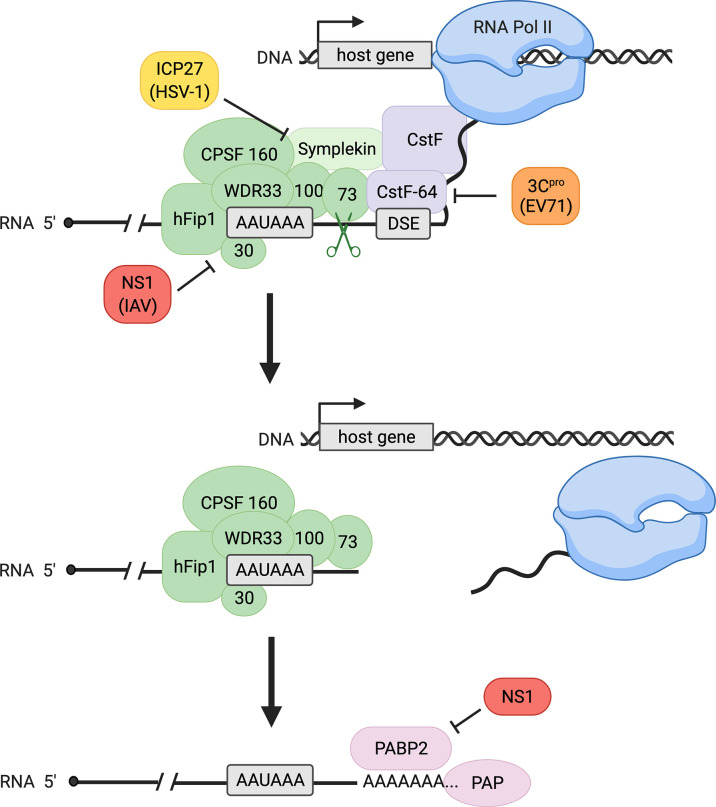
Canonical host 3´-end processing is a multi-step process with multiple opportunities for interference by viral proteins. RNA 3´-end processing begins with endonucleolytic cleavage of the nascent transcript by cleavage and polyadenylation specificity factor (CPSF, shown in green), composed of CPSF30, CPSF73, CPSF100, CPSF160, WDR33, and hFip1, and auxiliary factors including cleavage stimulation factor (CstF, a four-subunit factor shown in purple with only its CstF-64 subunit specified) and symplekin. The CPSF complex recognizes the polyadenylation signal (PAS, most common sequence AAUAAA as shown) on the transcript, and the catalytic subunit CPSF73 cleaves at a site downstream of the PAS, facilitated by CstF binding to the downstream sequence element (DSE). Following cleavage, transcription terminates, releasing the nascent RNA and RNA polymerase II (Pol II) from the DNA. Polyadenylation of the cleaved transcript is catalyzed by poly(A) polymerase (PAP) and supported by poly(A) binding protein II (PABP2). During viral infection, influenza A virus (IAV) protein NS1 binds CPSF30, preventing CPSF-RNA binding, whereas herpes simplex virus-1 (HSV-1) protein ICP27 inhibits proper assembly of the CPSF complex. Enterovirus 71 (EV71) 3C protease (3C^pro^) cleaves CstF-64, decreasing RNA 3´-end processing efficiency. Influenza NS1 also binds PABP2, inhibiting the synthesis of long poly(A) tails.

Transcription of eukaryotic genes continues until termination, marked by cleavage of the pre-mRNA and discontinuation of RNA synthesis by RNA polymerase II (Pol II). Termination of eukaryotic pre-mRNAs and 3´-end processing, which refers to the endonucleolytic cleavage process followed by polyadenylation (**Figure 1**), are closely coupled. Cleavage is executed by a protein complex termed cleavage and polyadenylation specificity factor (CPSF), whose subunits include CPSF30, CPSF73, CPSF100, CPSF160, WDR33, and hFip1. CPSF recognizes the polyadenylation signal (PAS), which is most commonly an AAUAAA sequence in mammals (1–6). Endonucleolytic cleavage occurs subsequently at a site 10-30 nucleotides downstream of the PAS and is catalyzed by the CPSF73 endonuclease (7, 8). In addition to CPSF, cleavage involves several other proteins including cleavage stimulation factor (CstF) in mammals (6). CstF-64, a critical subunit of the CstF complex, recognizes the downstream sequence element (DSE) (**Figure 1**). This element consists of a G/U- or U-rich region located up to 30 nucleotides downstream of the cleavage site and influences 3´ cleavage as well as 3´-polyadenylation efficiency after cleavage (1, 2). Importantly, 3´-end cleavage is required for transcription termination to occur: after the critical cleavage step, the 5´-3´ exonuclease Xrn2 is able to degrade the transcript that Pol II continues to synthesize downstream of the PAS until Xrn2 reaches the polymerase. This interaction causes Pol II to release the DNA, completing the process of termination (**Figure 1**) (9).

Polyadenylation is mediated by poly(A) polymerase (PAP), which catalyzes the addition of 50-100 adenine nucleotides to the exposed 3´OH of the mRNA after cleavage, forming the poly(A) tail (**Figure 1**) (2, 10). While poly(A) tail addition does not require a specific template sequence, the length of the tail is thought to be influenced by PAP, CPSF, and polyadenylate-binding nuclear protein 1 (PABPN1), also known as poly(A) binding protein II (PABP2) (2, 11). Cleavage and polyadenylation are tightly coupled and are regulated by many other factors and auxiliary elements (2). The polyadenylation of mRNAs is essential for subsequent translation, as the poly(A) tail influences mRNA stability, localization, and translational competency (2).

Disruption of cleavage and polyadenylation can have severe effects. Because of the importance of the PAS in 3´-end processing and consequently transcription termination, PAS mutations can significantly impact transcript levels. In turn, changes in the levels of critical mRNAs can cause severe disease. For example, PAS mutations in the *FOXP3* gene lead to downregulation of FOXP3, a key transcription factor for regulatory T cells, resulting in IPEX syndrome, a fatal autoimmune disorder linked to defective development of regulatory T cells (1, 12, 13). Deficits in the 3´-end processing machinery are also associated with cell-cycle arrest, apoptosis, and cancer/tumor formation (1, 13–15). Disruptions in poly(A) tail formation can lead to altered localization of mRNAs, potentially impeding translation of critical mRNAs into functional proteins (16, 17). The poly(A) tail further serves as a protective feature. Mutations in components of the polyadenylation machinery such as PAP can lead to decreased polyadenylation and to premature mRNA decay through intrinsic cell mechanisms such as nonsense-mediated decay (NMD) and degradation by the nuclear exosome (1, 2, 18–20).

Furthermore, transcription itself is tightly coupled to 3´-end processing: transcriptional activators influence 3´-end cleavage, and 3´-end processing is linked to transcription termination (9, 21, 22). This interaction between transcription and 3´-end processing factors occurs co-transcriptionally, indicating that the two processes are mechanistically linked (1, 2, 22–25). Alterations to one of these processes are expected to affect the other, presenting potential for dysregulation of translationally competent mRNA formation at both levels.

Viruses are known to cause global host shutoff, defined as suppression of cellular gene expression during infection. Thus, viruses can co-opt host transcriptional and translational machinery for viral gene expression, as well as reduce expression of host genes that may be detrimental to viral survival and proliferation (26, 27). Diverse mechanisms underlie these effects on host cells, including suppression of transcription through interaction of viral proteins with transcription factors and promoters, inhibition of Pol II elongation, and degradation of Pol II through ubiquitination (28–31). Viral infection also interferes with mRNA processing and stability through its effects on splicing, 5´ capping, and nuclear export of cellular mRNAs as well as induction of mRNA degradation (32–37). Many mechanisms for selectively expressing viral over host genes alter the population of mRNAs available for translation rather than destroying or inhibiting the translational machinery, thereby enabling the virus to continue to utilize host ribosomes to translate its own mRNAs (26). Overall, disruption of 3´-end processing and transcription termination reduces production and disrupts localization of translatable host mRNAs, making these processes appealing targets for a wide range of viruses as a strategy for host shutoff.

This review describes many of the known effects of certain viruses on RNA 3´-end processing and transcription termination, including read-through transcription induced by influenza A protein NS1 and herpes simplex virus-1 (HSV-1) protein ICP27. Read-through transcription causes production of Downstream-of-Gene (DoG) transcripts that are retained in the nucleus and thus not translated. Such genome-wide disruption of transcription termination affects thousands of genes, including those involved in the innate antiviral response. Remarkably, the ability of viruses to disrupt host 3´-end processing specifically, while preserving their own transcription and 3´-end processing, allows for efficient viral gene expression and simultaneous host shutdown.

## Viruses Disrupt 3´-end Processing *via* a Variety of Mechanisms

Viruses disrupt host mRNA processing using various mechanisms (32–35, 37). Notably, direct viral interference with mRNA transcription and maturation is not limited to a certain viral family or class (DNA/RNA, positive/negative sense), but occurs in a diverse range of viruses (28, 33). Although only a few viruses have been reported to manipulate host RNA 3´-end processing, this approach appears not to be restricted to a specific viral class.

### Influenza Virus Interferes With 3´-End Cleavage and Polyadenylation

Influenza virus is known to affect host RNA processing negatively through several mechanisms, including disruption of both 3´- and 5′-end processing (32, 38–41). At the 5´-end, the virus snatches a segment of the capped 5´-end of a nascent cellular RNA to prime viral transcripts and impede expression of many host genes through premature termination (32, 38). At the 3´-end, the virus inhibits transcription termination by interfering with cellular 3´-end cleavage (40, 41). Viral protein NS1 interacts directly with CPSF30 to prevent the CPSF complex binding to pre-mRNA (**Figure 1**). NS1 protein overexpression indeed yields higher levels of uncleaved mRNAs, confirming inhibition of 3´-end cleavage by the CPSF complex (40). This disruption of 3´-end cleavage during influenza infection causes defective RNA Pol II termination, resulting in the production of long read-through transcripts also known as DoG RNAs (41).

There is conflicting evidence regarding the necessity of NS1 in viral disruption of transcription termination. Read-through transcription also occurs independently of CPSF-NS1 interaction in influenza strains producing NS1 proteins that do not bind CPSF (41). Alternatively, NS1 has been reported to be necessary for the disruption of termination observed during influenza infection; a virus with an NS1 mutation lacking the ability to bind CPSF was able to replicate but did not cause increased production of read-through transcripts (42). This strain also displayed reduced virulence, suggesting that the NS1-CPSF interaction enhances the severity of influenza infection (42). However, it remains unclear how much of this attenuation of virulence can be attributed to the NS1-CPSF interaction, as NS1 is known to affect multiple cellular processes (43, 44). Furthermore, Hale et al. subsequently demonstrated that a recombinant strain of influenza in which NS1 is specifically mutated to bind CPSF30 optimally displays reduced virulence (45). Thus, it remains unclear how the interaction between CPSF30 and NS1 affects influenza virulence.

Additionally, NS1 interferes with polyadenylation through binding to PABP2 using a region distinct from that which binds CPSF. This interaction leads to nuclear redistribution of PABP2, as well as disruption of its nucleocytoplasmic shuttling, and a consequent decrease in polyadenylation of host transcripts (46).

### HSV-1 Infection Inhibits Cellular Transcription Termination

Infection with herpes simplex virus-1 (HSV-1) causes disruption of transcription termination in host genes *via* its immediate-early protein ICP27 (31, 47, 48). Deletion of ICP27 significantly reduces disruption of host termination, and ectopic expression of the protein is sufficient to inhibit RNA Pol II termination (48). While ICP27 deletion does not fully restore efficient transcription termination, Wang et al. describe a mechanism that convincingly suggests a role for the viral protein in disruption of 3´-end processing (48). ICP27 associates with several CPSF subunits, including CPSF30, CPSF73, CPSF100, CPSF160, and hFip1, but not with other cleavage and polyadenylation factors such as CstF-64 (**Figure 1**). Additionally, reduced binding of symplekin, a protein that typically associates with the 3´-end processing machinery, to CPSF was observed in the presence of ICP27 (48). These findings indicate that ICP27 may interfere with assembly of the canonical CPSF complex, leading to disruption of cleavage and polyadenylation. Since ICP27 alone did not fully recapitulate the extent of read-through transcription observed upon infection, the protein may not be solely responsible for HSV-induced read-through transcription (47, 48).

### Enterovirus 71 Infection Results in Decreased 3´-End Processing *via* Cleavage of CstF-64

Infection by enterovirus 71 (EV71), a picornavirus, also affects host transcription and 3´-end processing factors (49, 50). Weng et al. demonstrated that EV71 3C protease (3C^pro^) causes *in vitro* cleavage of CstF-64 (**Figure 1**) (50). In EV71-infected cells, the amount of CstF-64 detected by fluorescence microscopy decreased progressively. Furthermore, infected cells displayed increased levels of uncleaved host pre-mRNA and decreased levels of polyadenylated mRNA when compared to mock-infected cells, which could be rescued by addition of CstF-64. These observations indicate that EV71 infection inhibits canonical 3´-end processing of host mRNAs using a mechanism dependent on 3C^pro^ and CstF-64 (50). However, the genome-wide effects of 3C^pro^ inhibition of CstF-64 are yet to be characterized. Additionally, multiple picornavirus proteases, including enterovirus 3C^pro^, target many factors involved in transcription, nuclear export, and translation (51). This observation suggests that enterovirus could interfere with host mRNA and protein production at multiple steps of the pathway.

### HIV Tat Protein Causes Increased Expression of CPSF73 *In Vitro*

Human immunodeficiency virus-1 (HIV-1) Tat protein stimulates Pol II transcription of viral genes (52). Tat protein is also hypothesized to affect host cellular functions such as transcription and translation, leading to shutoff of critical host genes while increasing expression of those favoring the spread of the virus (53, 54). In a study by Calzado et al., HIV Tat overexpression caused specific upregulation of CPSF73 mRNA and protein expression, although whether Tat modulates CPSF expression in viral infection is not yet known (55). Rather than its known role in 3´-end processing, CPSF73 was found to interact with and suppress transcription at the HIV-1 LTR promoter, and transcription but not CPSF binding to the promoter was counteracted by HIV Tat (56). The potential role of Tat protein and HIV-1 infection in the regulation of RNA 3´-end processing remains to be elucidated. HIV also interacts with other cleavage factors: specifically, its capsid interacts with CPSF6, and this interaction plays a role in facilitating HIV genome integration into gene-dense regions of the host genome (57). However, potential impacts of this interaction on host 3´-end processing have not been clarified.

For other viruses, little has been reported regarding disruption of 3´-end processing, read-through transcription, and downstream effects of these processes during infection. However, other viruses use a multitude of methods to suppress host gene expression at several stages of the RNA production and maturation pathway, including interference with 5´-end processing and dysregulation of splicing (28, 33, 35). It is possible that disruption of 3´-end processing is more widespread than currently appreciated, but this question remains to be further explored.

## Host-Specific Disruption of Transcription Termination Is Widespread and Affects the Antiviral Response

The extent of host RNA processing defects in virus-infected cells had remained unclear as understanding the relevant viral mechanisms was initially limited to the examination of sample genes or the use of reporter constructs. More recently, with genome-wide analysis through next-generation sequencing, it has become evident that viral disruption of mRNA processing affects cellular genes globally across the transcriptome. For instance, 7-8 hours after HSV-1 infection, 64% of cellular genes experienced greater than 15% of their total transcripts reading through (47). In fact, 26% of genes displayed striking read-through levels of greater than 75%, highlighting the pervasive read-through transcription observed during viral infection (47).

In another example, during infection with an influenza strain (A/BM/1/18) that expresses NS1 expected to interact with CPSF, more than 600 host genes were downregulated (39). Importantly, increased readthrough was inversely related to the level of gene expression, and the majority of read-through genes in influenza infection experienced downregulation (39, 40). Notably, influenza viruses containing NS1 proteins with mutated CPSF binding sites displayed relief of inhibition of host mRNA production for several examined genes as well as restoration of mRNA 3´-end cleavage (40, 58, 59). These results collectively suggest that NS1 binding to CPSF and consequent disruption of host 3´-end processing and termination within the infected cell negatively regulates host gene expression on a global scale.

Viruses also inhibit the production of critical antiviral mRNAs through induction of read-through transcription, though it is not yet known whether specific immune pathways are selectively targeted. Salient genes in the innate antiviral response are affected. For example, in influenza strain A/Udorn/72, NS1 binding to CPSF30 inhibits 3´-end processing of the mRNA for IFN-β, a key regulator of the type I interferon response (42, 59). When compared to wild-type infection, a CPSF-binding mutant virus led to increased and earlier production of IFN-β mRNA, as well as mRNAs of two other key antiviral genes, ISG15 and MxA. Proliferation of the virus was significantly affected, as evidenced by inhibited viral replication upon mutation of the CPSF30 binding site on NS1 (42, 59).

In HSV-1 infection, the gene with highest level of readthrough is interferon regulatory factor 1 (IRF1) (60). IRF1 is a transcription factor in the interferon signaling pathway that directly binds to the promoters of and regulates the expression of many antiviral genes. Notably, as early as 2-3 hours post HSV-1 infection, greater than 75% of transcription from IRF1 was read through and did not generate mRNAs. This study did not find any functional enrichment for genes that are significantly read through in HSV-1 infection (60). However, the fact that critical immune regulator genes, including IFN-β and IRF1, are read through in virus-infected cells demonstrates that viruses can dampen the host antiviral response *via* manipulation of 3´-end processing to ensure successful infection of the host cell. Whether viruses preferentially suppress antiviral mRNA production *via* this mechanism remains to be further examined.

**Figure 2:**
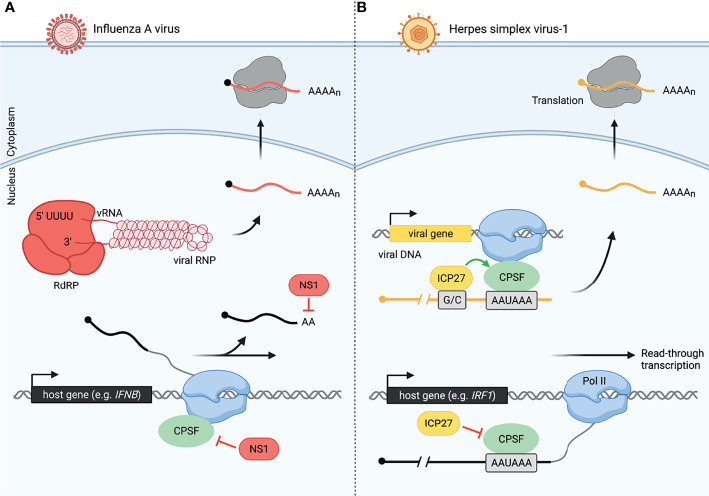
Viruses have evolved distinct mechanisms to modulate host and viral 3´-end processing, allowing host-specific disruption. **(A)** Influenza A virus (IAV) uses NS1 protein to disrupt cellular 3´-end processing and a distinct method for viral 3´-end processing. Inhibition of cleavage and polyadenylation by NS1 causes read-through transcription of many host genes including *IFNB* during influenza infection. Meanwhile, influenza produces an RNA-dependent RNA polymerase (RdRP), allowing it to transcribe and process viral RNA (vRNA, shown as part of the IAV viral ribonucleoprotein, vRNP). Viral mRNA is also polyadenylated by RdRP once the polymerase reaches a 5´-U track on the vRNA. These viral mRNAs are then exported to the cytoplasm for translation by host ribosomes. Influenza viral RNA and proteins are colored in red, and host RNA is colored in black. **(B)** Herpes simplex virus-1 (HSV-1) uses ICP27 protein to disrupt cellular 3´-end processing and promote viral 3´-end processing. CPSF is unable to assemble correctly in the presence of ICP27. This leads to read-through transcript production from thousands of host genes including *IRF1* in HSV-1-infected cells. HSV-1 uses the host machinery, RNA polymerase II (Pol II) and CPSF, for viral transcription and 3´-end processing, respectively. However, a GC-rich sequence (G/C) preceding the viral PAS is recognized by ICP27, allowing efficient viral pre-mRNA cleavage by CPSF. Herpes viral RNA and proteins are colored in yellow, and host RNA is colored in black.

Global analysis of read-through transcription has revealed that despite the extensive 3´-end processing defects in virus-infected cells, viruses evade disruption of 3´-end processing of their own transcripts (**Figure 2**). Influenza virus avoids interfering with 3´-end processing of its own mRNAs in a manner distinct from that of HSV-1. Influenza is a negative-sense RNA virus, which encodes an RNA-dependent RNA polymerase (RdRP) to produce viral mRNAs. RdRP elongates the viral mRNA using the viral RNA (vRNA) as a template until it reaches a 5´-U track in the vRNA, which serves as a signal and template for polyadenylation by RdRP, avoiding the use of host transcription and 3´-end processing machinery altogether (**Figure 2A**) (32, 61, 62). In contrast, HSV-1 is a double-stranded DNA virus that relies on the host transcription machinery and 3´-end processing complex (28, 31). Intriguingly, HSV-1 protein ICP27, while globally disrupting transcription termination, conversely facilitates RNA processing of HSV-1 mRNAs (48). It was observed that the distinction between sites at which ICP27 causes termination defects and those at which the effect is opposite depends on the GC content of about 1kb lying immediately upstream of the polyadenylation signal (PAS) (48). ICP27 binds the GC-rich sequence rather than the 3´-end processing complex, allowing canonical processing to occur (**Figure 2B**) (48, 63). The inverse relationship between upstream GC content and disruption of transcription termination versus a propensity for HSV-1 RNAs to have a significantly higher GC content upstream of the PAS suggests that the virus may be using ICP27 to reduce host 3´-end processing and increase viral mRNA processing simultaneously (48). Furthermore, it was observed that at late stages of HSV-1 infection, viral protein-coding genes represent 80% of both nascent mRNA transcription and translation (47). Thus, viruses not only evade virally-induced disruption of transcription, but can in fact harness host machinery to support viral gene expression.

## Read-Through Transcription Affects Host Transcripts in Diverse Ways

**Figure 3:**
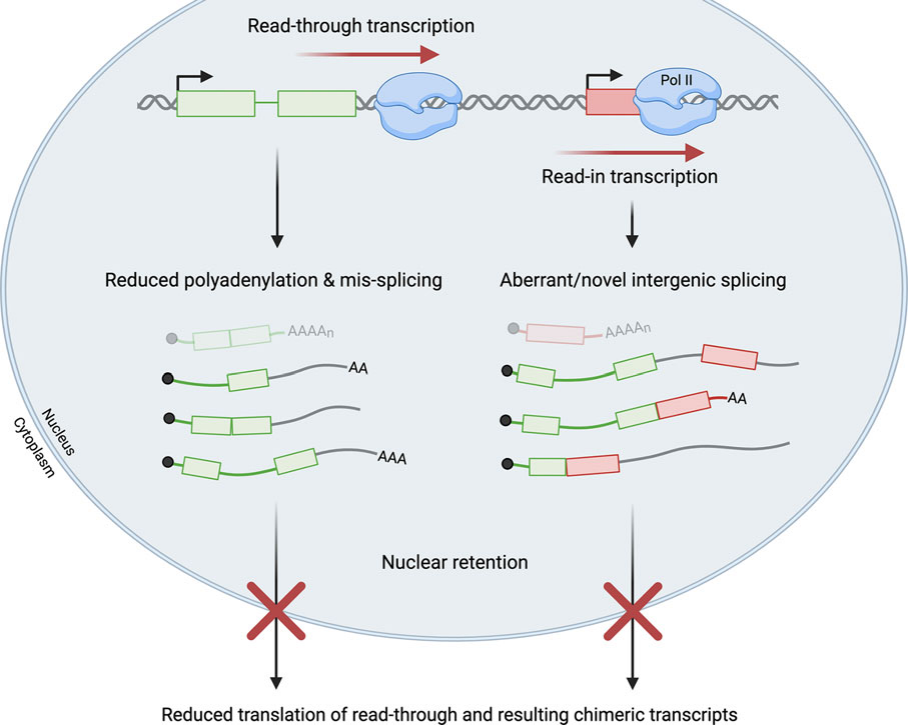
Read-through transcription has a multitude of effects. Failure of transcription termination and resultant read-through transcription can lead to read-in transcription of a downstream gene by RNA polymerase II (Pol II). Read-through transcripts are often mis-spliced and not polyadenylated. Read-in transcription is frequently associated with aberrant and novel intergenic splicing that generates chimeric transcripts. Read-through transcripts, including the subset of chimeric transcripts, are generally retained in the nucleus and not translated. In virus-infected cells, significant levels of read-through and read-in transcription impede the production of functional mRNAs (shown at the top in faded colors) from both the upstream read-through gene (shown in green with two exons) and the downstream read-in gene (shown in red with one exon). Green/red lines represent intronic or untranslated regions while grey lines represent intergenic sequences in read-through transcripts.

Viral infections promote failure of transcription termination at many cellular genes, but the roles of the act of read-through transcription versus the resulting transcripts remain unclear. Interestingly, production of read-through transcripts has also been observed in a variety of cellular stress conditions, including oxidative and osmotic stress (64–66). Only a few studies have directly compared the effects of read-through transcription in viral infection and cellular stress (41, 60). Collectively, these studies have revealed outcomes that are common to both stressed and virus-infected cells, as well as others that appear unique to virus-infected cells. Regardless of the cause, it is now evident that read-through transcripts are often not polyadenylated, are retained in the nucleus, and thus are non-coding (40, 47, 66). Notably, the fraction of transcripts that are read through from a given gene is significantly greater in virus-infected than in stressed cells, perhaps explaining why readthrough is strongly associated with reduced expression of the read-through gene during viral infection (31, 60). A similar response has not been clearly demonstrated in stressed cells (60). Both stress- and virus-induced read-through transcripts have been associated with splicing pattern changes in the upstream gene body region, supporting the notion that splicing and 3´-end processing regulate each other (60, 67–69). Readthrough can also result in read-in transcription of downstream genes in both virus-infected and stressed cells, whereby chimeric transcripts containing upstream and downstream sequences are generated (**Figure 3**). Read-in possibly but not necessarily leads to transcriptional interference with downstream gene expression (47, 60, 70, 71).

### Nuclear Retention of Transcripts

Influenza NS1 overexpression leads to a decrease in pre-mRNA cleavage, and the resulting read-through transcripts are polyadenylated at lower frequency than mRNAs in NS1-mutant overexpressing cells (40). The associated increase in nuclear retention of read-through transcripts was subsequently traced to NS1 interference with poly(A)-binding protein II (PABP2) and inhibition of the synthesis of long poly(A) tails, leading to reduced nuclear export of read-through transcripts (**Figure 3**) (46). Thus, this viral protein may affect several processes to ensure host shutoff at multiple levels.

In HSV-1-infected cells, Hennig et al. reported that read-through transcripts are enriched in nucleoplasmic and chromatin fractions as compared to the cytoplasmic fraction (60). Consistently, genes that were read through >75% upon HSV-1 infection displayed increased nucleoplasmic enrichment of the corresponding transcripts after infection, while genes that were not read through did not show changes in enrichment (60, 64). Read-through transcripts generated during cellular stress conditions are also retained in the nucleus (64).

### Reduced Translation and Protein Yield

Transcriptional readthrough during HSV-1 infection is both pervasive and potent, affecting many host genes to a great extent. Specifically, by 8 hours post infection (h.p.i.), 53% of all cellular genes experienced greater than 35% readthrough, whereas the median read-through levels at peak induction were 6-15% in cells subject to hyperosmolarity or heat shock (47, 60). Consequent gene expression changes associated with readthrough are significant in HSV-infected cells. Cellular genes experiencing >35% readthrough displayed a more marked decline in translation over the course of infection than those experiencing ≤5% readthrough (47). For example, ribosome profiling revealed that translation of the IRF1 mRNA, whose gene was read through >75%, decreased more than 4-fold at 8 h.p.i despite an increase in total level of IRF1 transcripts, coinciding with a 4-6-fold increase in nuclear enrichment of the IRF1 transcript after infection. This finding reinforces the notion that read-through transcripts are not available for translation (**Figure 3**) (60).

In the case of influenza virus infection, it was also proposed that host genes with decreased protein production were read through at high levels and that termination failure contributed to their low expression (39). Readthrough was also detected for genes that were upregulated, including interferon-stimulated genes such as IFIT1 and IFIT2 (39). We suspect that the increased levels of total transcripts reflect increased levels of non-coding DoG transcripts rather than of functional mRNA production, as in the case of IRF1 in HSV-1 infection. Importantly, viral infection leads to a higher extent of read-through transcription than do other cellular stress conditions (60). This could explain why reduced translation of read-through genes has thus far been observed only in viral infection. Indeed, the potential for readthrough to affect gene expression may be correlated with the severity of the disruption of termination. This relationship during influenza infection remains to be further dissected and is critical due to the observed readthrough of many cellular genes. Such experiments will aid in developing a better understanding of the underlying mechanisms by which influenza virus affects host gene expression and the contribution of read-through transcription to these observations.

### Mis-Splicing of Read-Through Transcripts

Read-through transcription is also associated with alterations to canonical splicing patterns in virus-infected cells. This includes aberrant splicing patterns in the gene body region upstream of the PAS at which readthrough occurs, as well as the downstream-of-gene regions that are read through (**Figure 3**).

After infection by influenza virus, defective RNA Pol II termination was observed 6 h.p.i., preceding genome-wide splicing deficits which were observable only 12 h.p.i., though splicing deficits of read-through transcripts in particular were not examined (39). If a causal relationship between these events could be demonstrated, it would suggest that inhibition of termination prevents the infected cell from producing viable transcripts through interference with a different step of RNA processing or vice versa. In other stress conditions, such as heat shock, widespread splicing deficits contribute to altering the cellular transcriptome, suggesting that aberrant splicing may serve to alter the pool of cellular transcription products that are viable for translation (72).

However, viral infection and other stress conditions appear to affect splicing of cellular RNAs in different ways: during viral infection, abnormal patterns of splicing have been observed and characterized, whereas heat shock leads to dramatic splicing inhibition and consequent intron retention and accumulation of pre-mRNAs in the cell nucleus (47, 72). HSV-1 infection induced splice junctions that are markedly enriched in transcripts with >35% readthrough compared to genes with <5% readthrough. Enriched junctions included those upstream of poly(A) sites at which readthrough occurred, as well as intergenic junctions between read-through and adjacent read-in genes (47). During heat shock, thousands of transcripts experience significant intron retention and nuclear localization, although whether stress-induced read-through transcripts are spliced inefficiently needs further examination (72). While canonical splicing activity is disrupted in both heat stress and HSV-1 infection, differences in the observed alterations of splicing patterns may reflect different goals. For example, widespread heat stress-related inhibition of splicing may focus cellular energy and resources on production of proteins needed for the stress response (72). In viral infection, abnormal splice junction usage may contribute to evasion of the antiviral response or other host-cell processes, such as promoting viral replication by increasing templates for cap-snatching or simply by decreasing the number of host transcripts exported to the cytoplasm (38).

During HSV-1 infection, read-through transcription is also accompanied by a significant increase in novel splice junctions not part of any annotated transcript, with at least 11% resulting from intergenic splicing between exons of two adjacent genes. Aberrant intergenic splicing was more prevalent in HSV-1-infected cells compared to cells experiencing hyperosmotic stress (60). This finding can likely be attributed to the significantly higher extent of read-through transcription and the longer length of the read-through transcripts produced during HSV-1 infection than during cellular stress (31). Further work is needed to elucidate the relationship between read-through transcription and changes in splicing events.

### Read-In Transcription

Read-through transcription can continue into downstream genes, a process known as read-in transcription, such that read-in genes are transcribed from the promoter of the upstream gene (47). At 7-8 hours post HSV-1 infection, at least 32.6% of all host genes displayed read-in transcript levels that accounted for more than 15% of total transcript levels of the read-in gene (47). Ribosome profiling revealed that of the open reading frames transcribed with significant (at least 35%) read-in transcription, 99% were not translated. Splicing was affected differently in different read-in transcripts, with almost no splicing observed for multi-exon long intergenic non-coding RNAs and splicing occurring at about 50% for protein-coding transcripts (47).

These observations emphasize the potential of read-through transcription to dysregulate gene expression at multiple critical steps. Preliminarily, we can conclude that read-through transcription may negatively regulate expression of both upstream and downstream genes. The fact that antiviral genes like IRF1 are affected implicates read-through transcription as a mechanism used by viruses to modulate the expression of important host genes and thereby to co-opt host-protein machinery for the production of viral proteins. Future work is needed to clarify the molecular mechanisms and effects of virally-induced host read-through transcription, including comparison of the differing strategies used by different viruses.

## Summary/Outlook

Viruses broadly disrupt host cellular processes upon infection, including those of the central dogma. Certain viruses, the best characterized being influenza A and HSV-1, disrupt host 3´-end processing and consequently transcription termination, leading to the production of read-through transcripts. The mechanisms by which various viruses disrupt termination appear to differ, but read-through transcription is clearly not limited to a certain class or family of viruses. Read-through transcription is important for host shutdown and for co-opting the host-cell machinery to utilize for viral gene expression. Supporting these ideas is the genome-wide nature of read-through transcription, which broadly affects thousands of host genes but spares viral genes. The diverse effects of read-through transcription on host gene expression include altered splicing, nuclear retention, and reduced translation of host transcripts, as well as read-in transcription of downstream genes. Further work is needed to characterize the effects of virally-induced read-through transcription on certain subsets of host genes such as antiviral genes and to understand the full range of viruses that exert these effects on cellular processes. Existing work provides a foundation for understanding virus-induced disruption of 3´-end processing in the larger scheme of viral infection and the antiviral response.

## Author Contributions

AV and AP are co-first authors, who with JS conceived the idea and wrote the manuscript. All authors contributed to the article and approved the submitted version.

## Funding

This work is supported by National Institutes of Health (NIH) grants R01GM140735 (to JS) and F30AI157301 (to AP). JS was supported by the Howard Hughes Medical Institute.

## Conflict of Interest

The authors declare that the research was conducted in the absence of any commercial or financial relationships that could be construed as a potential conflict of interest.

## Publisher’s Note

All claims expressed in this article are solely those of the authors and do not necessarily represent those of their affiliated organizations, or those of the publisher, the editors and the reviewers. Any product that may be evaluated in this article, or claim that may be made by its manufacturer, is not guaranteed or endorsed by the publisher.
